# Abdominal obesity is a more important causal risk factor for pancreatic cancer than overall obesity

**DOI:** 10.1038/s41431-023-01301-3

**Published:** 2023-05-10

**Authors:** Jared G. Maina, Vincent Pascat, Liudmila Zudina, Anna Ulrich, Igor Pupko, Amélie Bonnefond, Zhanna Balkhiyarova, Marika Kaakinen, Philippe Froguel, Inga Prokopenko

**Affiliations:** 1 INSERM UMR 1283, CNRS UMR 8199, European Genomic Institute for Diabetes (EGID), Institut Pasteur de Lille, F-59000 Lille, France; 2 University of Lille, Lille University Hospital, Lille, F-59000 France; 3 Section of Statistical Multi-Omics, Department of Clinical and Experimental Medicine, School of Biosciences and Medicine, University of Surrey, Guildford, UK; 4 Department of Metabolism, Digestion and Reproduction, Imperial College London, London, UK; 5 People-Centred Artificial Intelligence Institute, University of Surrey, Guildford, UK

**Keywords:** Obesity, Genome-wide association studies

## Abstract

Obesity and type 2 diabetes (T2D) are associated with increased risk of pancreatic cancer. Here we assessed the relationship between pancreatic cancer and two distinct measures of obesity, namely total adiposity, using BMI, versus abdominal adiposity, using BMI adjusted waist-to-hip ratio (WHRadjBMI) by utilising polygenic scores (PGS) and Mendelian randomisation (MR) analyses. We constructed z-score weighted PGS for BMI and WHRadjBMI using publicly available data and tested for their association with pancreatic cancer defined in UK biobank (UKBB). Using publicly available summary statistics, we then performed bi-directional MR analyses between the two obesity traits and pancreatic cancer. PGS_BMI_ was significantly (multiple testing-corrected) associated with pancreatic cancer (OR[95%CI] = 1.0804[1.025–1.14], *P* = 0.0037). The significance of association declined after T2D adjustment (OR[95%CI] = 1.073[1.018–1.13], *P* = 0.00904). PGS_WHRadjBMI_ association with pancreatic cancer was at the margin of statistical significance (OR[95%CI] = 1.047[0.99–1.104], *P* = 0.086). T2D adjustment effectively lost any suggestive association of PGS_WHRadjBMI_ with pancreatic cancer (OR[95%CI] = 1.039[0.99–1.097], *P* = 0.14). MR analyses showed a nominally significant causal effect of WHRadjBMI on pancreatic cancer (OR[95%CI] = 1.00095[1.00011–1.0018], *P* = 0.027) but not for BMI on pancreatic cancer. Overall, we show that abdominal adiposity measured using WHRadjBMI, may be a more important causal risk factor for pancreatic cancer compared to total adiposity, with T2D being a potential driver of this relationship.

## Introduction

Pancreatic cancer is a rare form of cancer, associated with poor prognosis and low survival rates [1]. Furthermore, epidemiological evidence from observational studies suggests obesity and type 2 diabetes (T2D) are major risk factors for pancreatic cancer [2, 3]. Body mass index (BMI) and waist-to-hip ratio (WHR) are two common metrics used to assess total and abdominal adiposity. However, despite being a routine measure of adiposity in clinical and research settings, BMI is an imperfect measure of metabolic health. Alternatively, WHR represents abdominal adiposity which has a stronger correlation to the metabolic syndrome compared to total adiposity [4]. To date, only 22 genome-wide significant signals are established in genome-wide association studies (GWAS) for pancreatic cancer [5]. In contrast, more than 600 and 300 signals have been reported for BMI and WHR, respectively [6, 7]. These individual associations from GWAS, however, do not explain the shared co-morbidity between obesity and pancreatic cancer. Nevertheless, genomic loci identified in GWAS could be implemented in methods such as polygenic scores (PGS) [8] and Mendelian randomization (MR) [9]. PGS can be used to define the shared genetic component between epidemiologically related phenotypes, while MR uses genetic variants as instruments to assess causality in relationships between phenotypes. In the present study, the impact of total and abdominal adiposity on pancreatic cancer risk was examined through PGS analyses, using publicly available GWAS of obesity traits data and information about pancreatic cancer within UK biobank. Moreover, using established genetic variants, we conducted a bi-directional MR between two adiposity traits and pancreatic cancer to assess the causal relationships between them.

## Materials and methods

### UK Biobank

The UK Biobank (UKBB) resource (www.ukbiobank.ac.uk) was used to define adiposity and cancer phenotypes for this study. We used the BMI data collected at the time of recruitment (UKBB field 21001). WHR data were computed by dividing waist circumference (UKBB field 48) by hip circumference (UKBB field 49) measured at baseline. BMI and WHR data were available for 457,270 individuals (Supplementary Fig. 1). For pancreatic cancer, we used a combination of hospital admissions data, the tenth revision of the International Classification of Disease (ICD-10) codes and self-report data. Individuals with an ICD-10 code (code C25) and who self-reported to have a pancreatic cancer diagnosis (code 1026) were set as cases, while individuals with no cancer diagnosis were set as controls. In total, there were 1416 cases and 455,854 controls (*n* = 457,270) for pancreatic cancer. To limit confounding by ancestry, only individuals of European ancestry were included in our analyses (Supplementary Methods, Supplementary Fig. 1).

### UKBB GWAS

We performed single phenotype GWAS in UKBB using the BOLT-LMM software [10]. BOLT-LMM applies a linear mixed model while age, sex, genotyping array and six principal components (PCs) were used as covariates for pancreatic cancer and BMI. BMI was an extra covariate in WHR GWAS to obtain WHRadjBMI analyses. The statistical threshold for genome-wide significant SNPs used was *P* < 5 × 10^−8^.

### Genetic correlation estimation

To estimate the genetic correlation (rG) between adiposity phenotypes (BMI/WHRadjBMI), T2D (Supplementary Methods), and pancreatic cancer in UKBB, we used the linkage disequilibrium (LD) score (LDSC) regression approach and tool [11].

### Polygenic scores

To construct BMI and WHRadjBMI PGS, we used risk-increasing alleles at 567 and 274 SNPs, respectively. The SNP list was obtained from recent large-scale GWAS meta-analyses by GIANT consortium [6, 7]. However, as the target data for PGS analysis was the UKBB, which was part of the GIANT meta-analyses, we used weights from the study which did not include UKBB in the meta-analyses [12, 13] (Supplementary Fig. 2). We used the PLINK software [14] to generate the PGS. We used sex, age, genotyping array and six PCs as covariates in the regression model. As a sensitivity analysis, we ran a regression model with T2D as an extra covariate.

### Mendelian randomization

To assess causality between the two adiposity measures and pancreatic cancer, we performed bi-directional MR using the *TwoSampleMR* R package [15]. We obtained the genetic instrument for BMI (566 SNPs) and WHRadjBMI (278 SNPs) from the GIANT consortium [6, 7]. The genetic instruments for pancreatic cancer (16 SNPs) were obtained from Klein et al [5]. The causal effect estimate was derived from the inverse-variance weighted (IVW) method [16]. The MR-Egger, simple mode, weighted mode and weighted median tests were used as sensitivity analyses [17]. We excluded palindromic SNPs from the exposure-outcome pairs and matched alleles between summary statistics as part of the *TwoSampleMR* pipeline. Outliers were removed after inspection of scatter plots and leave-one-out results. Heterogeneity among the genetic instruments was evaluated using Cochran’s Q test.

## Results

### UKBB GWAS and genetic correlation estimates

**Fig. 1:**
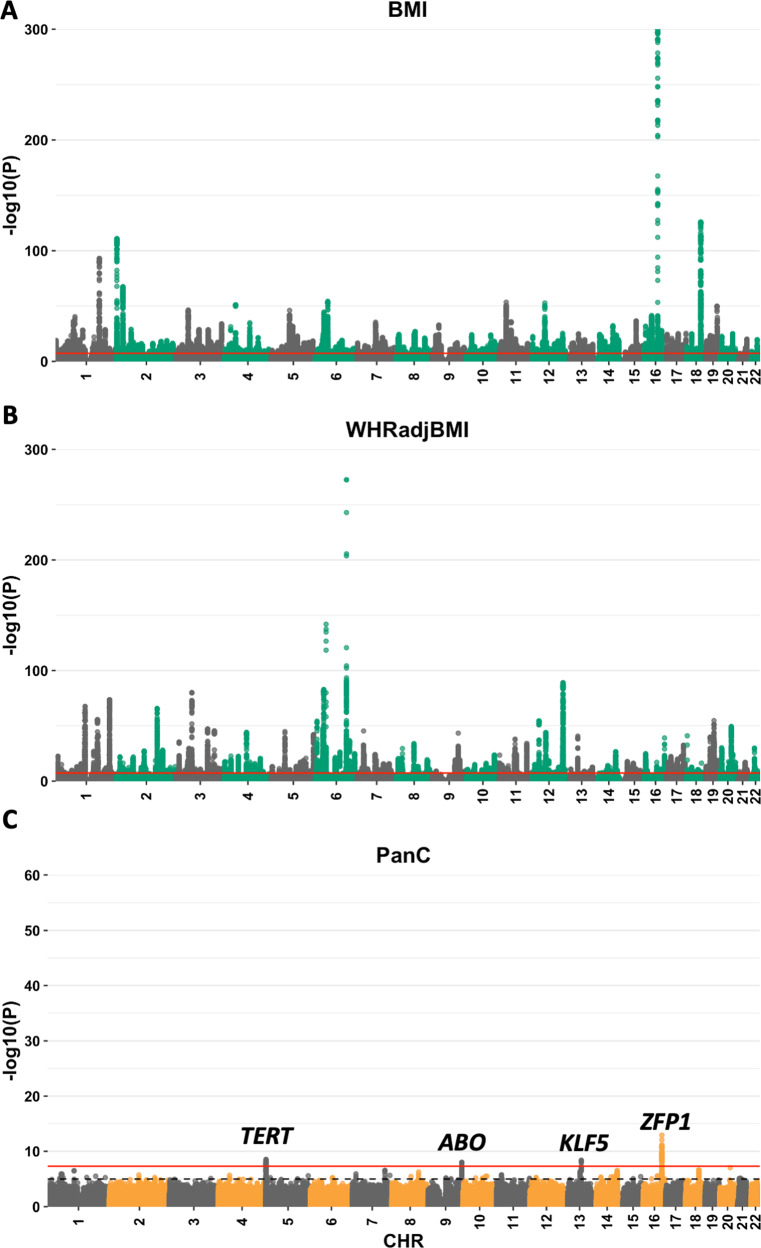
UK Biobank GWAS results. Manhattan plots of **A** BMI, **B** WHRadjBMI, and **C** pancreatic cancer GWAS in UK Biobank. The red horizontal line shows genome-wide significance threshold (*P* < 5 × 10^−8^). The dashed grey line shows suggestive significance threshold (*P* < 1 × 10^−5^).

In UKBB GWAS, we identified 998, 1014 and 4 significant independent SNPs at 901, 718, 4 *loci* for BMI, WHRadjBMI and pancreatic cancer respectively (Fig. 1). The four *loci* identified for pancreatic cancer were *TERT*, *ABO, KLF* and *ZFP1* (Fig. 1C) in line with recently published GWAS of pancreatic cancer [5]. None of the obesity signals were shared with pancreatic cancer in the UKBB. However, 3 of the 22 established pancreatic cancer loci by Klein et al. [5] were shared with WHRadjBMI in UKBB and had same direction of effect. These were *NR5A2*, *ETAA1* and *ZNRF3*. Conversely, only *ETAA1* from Klein et al. [5] was shared with BMI in the UKBB. Additionally, there was positive genetic correlation between both obesity measures and pancreatic cancer, but the estimates did not meet statistical significance (rG_BMI_ = 0.472, *P* = 0.479, rG_WHRadjBMI_ = 0.098, *P* = 0.671) (Supplementary Table 1). Similarly, the genetic correlation between T2D and pancreatic cancer in the UKBB was underpowered and did not meet statistical significance (rG = −0.0139, *P* = 0.961) (Supplementary Table 1).

### Effects of obesity variants on pancreatic cancer via polygenic scores

We identified a significant (Bonferroni multiple testing corrected *P* = 0.05/2 tests = 0.025) direct association between BMI PGS and pancreatic cancer (OR[95%CI] = 1.0804[1.025–1.14], *P* = 0.0037). We also identified a direct association between WHRadjBMI PGS and pancreatic cancer, however, this association was not statistically significant (OR[95%CI] = 1.047[0.99–1.104], *P* = 0.086) (Table 1). To determine if the association between adiposity PGS and pancreatic cancer was driven by T2D, we adjusted for T2D in the association tests. After T2D adjustment, the significance of the association for both BMI and WHRadjBMI PGS declined to suggest that T2D could be acting via adiposity in pancreatic cancer risk (OR_BMI_PGS_[95%CI] = 1.073[1.018–1.13], *P* = 0.00904); OR_WHRadjBMI_PGS_[95%CI] = 1.039[0.99–1.097], *P* = 0.14). Notably, the decline in association after T2D adjustment was more for WHRadjBMI PGS than BMI PGS (Table 1).

**Table 1:** Association between adiposity polygenic scores and pancreatic cancer.

	Unadjusted model		T2D adjusted model	
Adiposity trait	OR (95%CI)	*P*	OR (95%CI)	*P*
BMI	1.0804 (1.025–1.14)	0.0037	1.073 (1.018–1.13)	0.00904
WHRadjBMI	1.047 (0.99–1.104)	0.086	1.039 (0.99–1.097)	0.14

*OR(95%CI)* = Odds ratio of association and the lower and upper 95% confidence intervals (CI).

### Causality results using Mendelian randomization

**Fig. 2:**
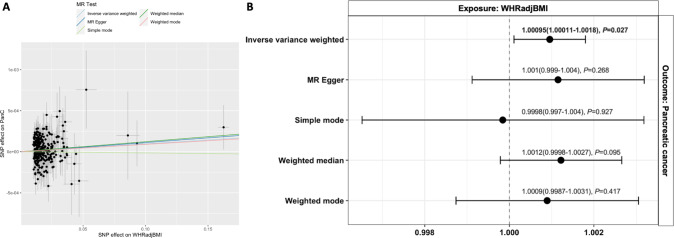
Abdominal obesity MR results. **A** Scatter and **B** forest plots for the WHRadjBMI to pancreatic cancer MR test. The scatter plot includes the intercepts of the various MR methods used while the odds ratio plot shows the MR effect estimate for each MR method used.

We report a causal effect of WHRadjBMI on pancreatic cancer at nominal significance (OR[95%CI] = 1.00095[1.00011–1.0018], *P* = 0.027) based on the IVW method, indicating a weak but positive causal effect estimate (Fig. 2). However, none of the other MR tests for this direction were significant. The Cochran’s Q test indicated the absence of heterogeneity among the genetic instruments (*Q*_IVW_ = 258.08, *P* = 0.787). On the contrary, we have not identified any causal effect (Bonferroni *P* = 0.05/4 tests = 0.0125) of BMI on pancreatic cancer in either of the MR tests performed (Supplementary Table 2). There was no evidence of a causal effect from pancreatic cancer to WHRadjBMI (OR_IVW_(*P*) = 0.143(0.604). The results from pancreatic cancer to BMI were less informative with large standard errors despite nominal significance in some of the sensitivity MR tests (OR_WeightedMedian_[95%CI] = 58.105[3.997–844.69], *P* = 0.003) (Supplementary Table 2).

## Discussion

In this study, using large-scale datasets and a multi-method approach, we show that abdominal obesity assessed using WHRadjBMI is a causal risk factor for pancreatic cancer, in line with epidemiological evidence [18].

The mechanisms underlying the obesity-pancreatic cancer co-morbidity remain unclear. However, several factors such as inflammation, insulin resistance and hyperinsulinemia are potential mechanisms linking obesity to cancers including that of the pancreas [3, 19]. Notably, majority of these factors are hallmarks of metabolic syndrome which correlate with abdominal obesity [20]. Therefore, it is not surprising that our Mendelian randomisation results show that WHRadjBMI rather than BMI is a more important causal risk factor for pancreatic cancer. Furthermore, the metabolic syndrome is considered a predictor of T2D [21]. In our polygenic score analyses, we show that after adjusting for T2D status, the significance of the association declined modestly for PGS_BMI_ while any evidence of association in PGS_WHRadjBMI_ on pancreatic cancer risk was effectively lost. Taken together, our polygenic analyses and Mendelian randomization suggest that the metabolic syndrome proxied by abdominal obesity may be a causal risk factor for pancreatic cancer. Additionally, obesity-associated T2D [22] may be a potential cause driver of the metabolic syndrome underlying pancreatic cancer progression in obesity [3].

Several limitations in our present studies should be considered. Pancreatic cancer is a rare form of cancer characterised by low sample sizes as compared to other more common cancers. Consequently, there is less power in GWAS to identify genetic loci amenable for statistical analyses. Additionally, the causal effect identified in MR is only nominally significant and therefore interpretation of our findings should consider this. Future work will focus on validating our results in larger datasets, especially for pancreatic cancer to improve statistical power of the analyses. Moreover, further analyses to properly control for T2D would be needed due to the complex relationship between obesity and T2D, more so in Mendelian randomization. Additional analyses to include components of the metabolic dysfunction such as fasting glucose levels will be part of future direction of this effort.

In conclusion, we show that abdominal adiposity measured through WHRadjBMI, may be a more important risk factor for pancreatic cancer, compared to total adiposity. Our results highlight the relationship between the metabolic syndrome component and a higher risk for pancreatic cancer, with T2D being a potential driver of this association. Furthermore, we demonstrate the importance and therefore encourage the assessment of diverse measures of obesity in clinical practice and research in the context of pancreatic cancer risk. Additionally, healthcare providers should emphasise the need for patients to monitor their visceral weight gain and not just overall weight gain to minimise the risk for pancreatic cancer.

## Supplementary information

Revised supplementary file

## Data Availability

The datasets generated and/or analysed during the current study are available from the corresponding author on reasonable request.
