# Supplementary material for: Abdominal obesity is a more important causal risk factor for pancreatic cancer than overall obesity
Source: Eur J Hum Genet. 2023 May 10;31(8):962–6. doi: 10.1038/s41431-023-01301-3 (PMC10400602; doi:10.1038/s41431-023-01301-3)
Supplement: Supplementary file 1 — Revised supplementary file [file 41431_2023_1301_MOESM1_ESM.docx]

**Supplementary Data – Abdominal obesity, rather than overall obesity, is a causal risk factor for pancreatic cancer**

**Supplementary Methods.**

Pancreatic cancer definition in UK Biobank (UKBB)

Pancreatic cancer in UKBB was defined using a combination of the tenth revision of the International Classification of Disease (ICD-10) codes and self-report data. Additionally, hospital admissions data, recently made available to researchers by UKBB, were used to supplement the number of cases. Individuals with an ICD-10 code (C25) and those who self-reported to have a pancreatic cancer diagnosis (code 1026) were set as cases. In total, there were 629 cases and 458,987 controls of European ancestry for pancreatic cancers after exclusions (**Supplementary Figure 1**). 1,340 European pancreatic cancer cases were defined from hospital admissions data (ICD-10 code C25). 544 of these cases were shared with the 629 cases defined using ICD-10 and self-report data only. 85 cases (self-reported) from the 629 cases defined earlier were added to the 1340 hospital admissions cases. 796 controls which had case status in the hospital admissions data were excluded from controls. In total, after all exclusions were applied, there were 1,416 cases and 455,854 controls of European ancestry for pancreatic cancer (**Supplementary Figure 1**).


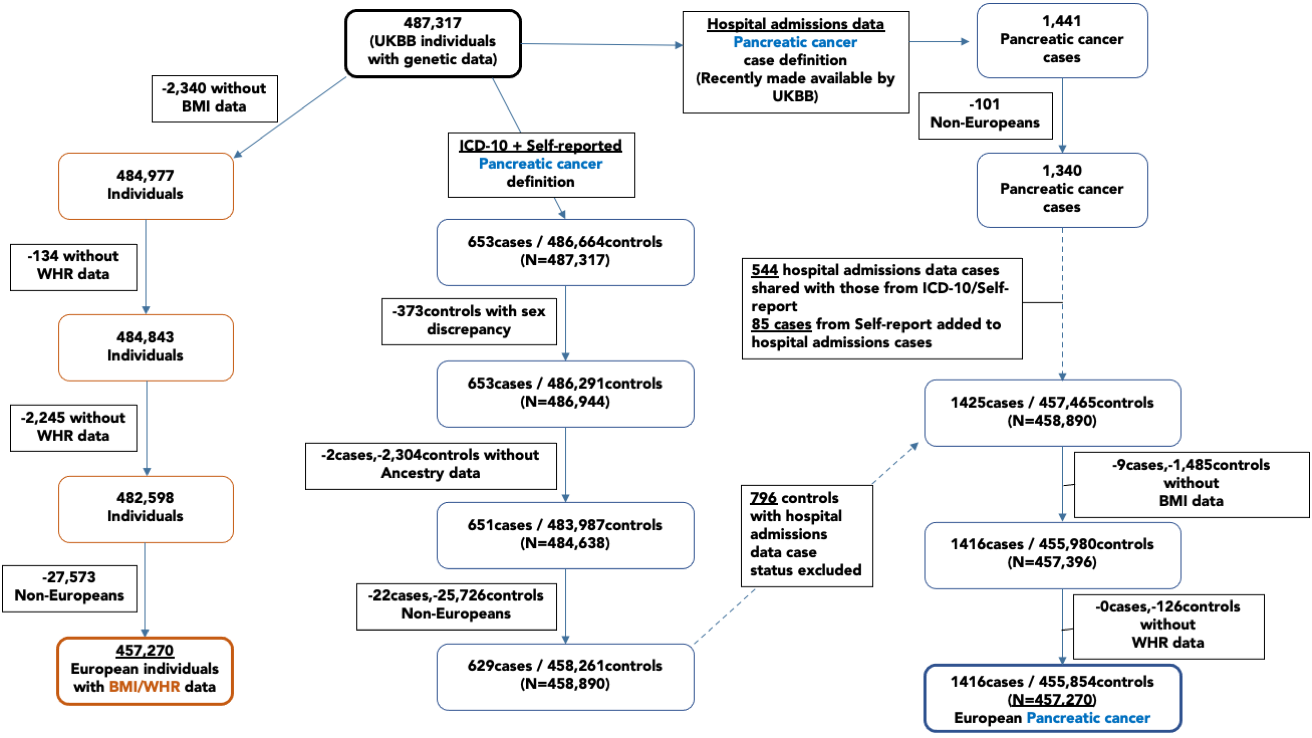


**Supplementary Figure 1**. Flow chart showing UK Biobank adiposity and pancreatic cancer definition

Type 2 diabetes definition in UKBB

To determine the role of type 2 diabetes (T2D) in the relationship between obesity and pancreatic cancer, we sought to first define the genetic correlation between T2D and pancreatic cancer. Secondly, we included T2D as an additional covariate in our polygenic scores (PGS) analyses. A T2D case in UKBB was defined if a participant self-reported a diabetes diagnosis made by a doctor, were on insulin medication one year post-diagnosis and were at least 40 years old by the time the diagnosis was made. T2D controls included individuals who did not meet the case criterion. From both cases and controls, we excluded individuals with gestational diabetes (UKBB field 4041, code=1), individuals on insulin medication within the first year of diagnosis (UKBB field 2986) and individuals who were younger that 40 years old at the time of diagnosis (UKBB field 2976). In total, we had 19,344 cases and 463,641 controls of European ancestry.

Genetic correlation estimation in UKBB

We used the LDSC regression tool^1^ to estimate the genetic correlation between BMI, WHRadjBMI and pancreatic cancer in UKBB. UKBB GWAS summary statistics were filtered based on the following parameters: imputation score > 0.9, minor allele frequency (MAF) > 0.01 and 0.1 ≥ P-value > 0. Strand ambiguous, duplicated SNPs and variants that did not represent SNPs (e.g., indels) were removed. The Bonferroni corrected significance threshold to determine significant genetic correlation estimates was set as P<0.025 (0.05/2, the number of genetic correlation tests done in our analyses; one for BMI and one for WHRadjBMI). Nominal significance threshold was set at 0.05 ≥ P > 0.025.

Polygenic scores

The SNP lists for BMI and WHRadjBMI were obtained from GIANT consortium’s meta-analyses^2,3^. The meta-analyses included previous GIANT studies^4,5^ and UKBB. Since we used the UKBB as the target data (testing cohort for our PGS), we use the weights from the studies that did not include UKBB in the meta-analyses. The workflow for PGS analyses is shown in **Supplementary Figure 2** below.


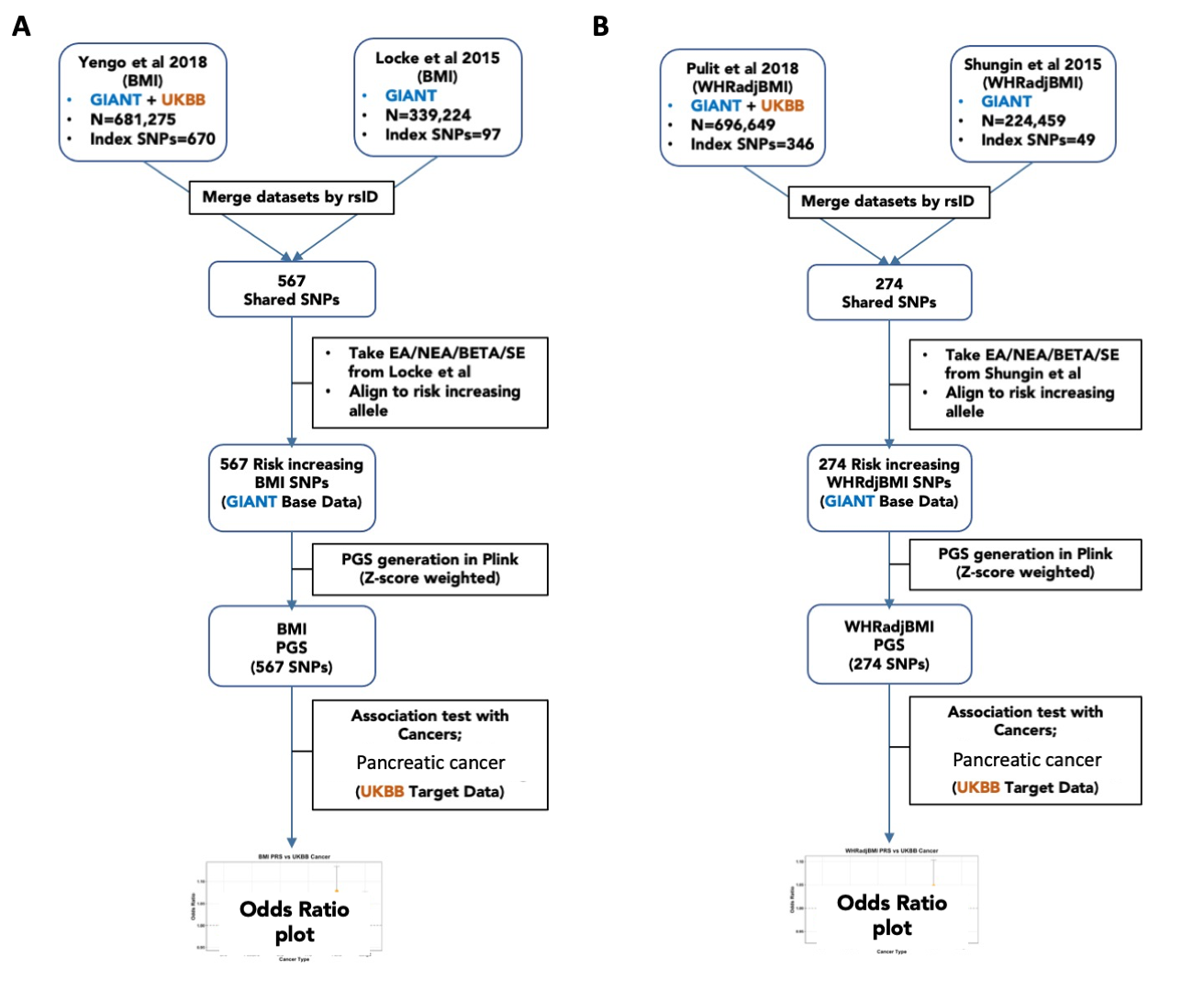


**Supplementary Figure 2**. (A) BMI and (B) WHRadjBMI polygenic score analyses pipeline. A two-sample approach was used to construct our PGS base data. The SNPs used for the PGS were from GIANT’s latest BMI and WHRadjBMI meta-analyses. Since the meta-analyses comprised of the UK Biobank (our target data), we used weights from the non-UK Biobank study in GIANT’s meta-analyses.

Mendelian randomization (MR)

We assessed the causal relationships between BMI, WHRadjBMI and pancreatic cancer using bi-directional MR. The *TwoSampleMR* R package^6^ was used for this analysis. We tested the effect of obesity (BMI and WHRadjBMI) as an exposure for pancreatic cancer (outcome), and the reverse direction with pancreatic cancer as a risk factor for obesity (BMI and WHRadjBMI) using summary statistics from independent datasets. The genetic instruments for BMI (670 SNPs) and WHRadjBMI (346 SNPs) were obtained from GIANT consortium^2,3^. Additionally, the pancreatic cancer genetic instruments (22 SNPs) were obtained from a recent large-scale meta-analysis by Klein et al^7^.

REFERENCES:

1. Bulik-Sullivan, B. *et al.* LD Score regression distinguishes confounding from polygenicity in genome-wide association studies. *Nature Genetics 2015 47:3* **47**, 291–295 (2015).

2. Yengo, L. *et al.* Meta-analysis of genome-wide association studies for height and body mass index in ∼700000 individuals of European ancestry. *Human Molecular Genetics* **27**, 3641–3649 (2018).

3. Pulit, S. L. *et al.* Meta-analysis of genome-wide association studies  for body fat distribution in 694 649 individuals of European ancestry. *Human Molecular Genetics* **28**, 166–174 (2019).

4. Locke, A. E. *et al.* Genetic studies of body mass index yield new insights for obesity biology. *Nature* (2015) doi:10.1038/nature14177.

5. Shungin, D. *et al.* New genetic loci link adipose and insulin biology to body fat distribution. *Nature 2015 518:7538* **518**, 187–196 (2015).

6. Hemani, G. *et al.* The MR-base platform supports systematic causal inference across the human phenome. *Elife* **7**, (2018).

7. Klein, A. P. *et al.* Genome-wide meta-analysis identifies five new susceptibility loci for pancreatic cancer. *Nature Communications 2018 9:1* **9**, 1–11 (2018).

**Supplementary Tables**

**Supplementary Table 1**. Genetic correlation results between adiposity measures, type 2 diabetes and pancreatic cancer in UKBB

|  | **BMI** | | | **WHRadjBMI** | | | **Type 2 diabetes** | | |
| --- | --- | --- | --- | --- | --- | --- | --- | --- | --- |
| **Cancer** | **rG**  **(SE)** | **Z score** | **P** | **rG**  **(SE)** | **Z score** | **P** | **rG**  **(SE)** | **Z**  **Score** | **P** |
| Pancreatic | 0.472  (0.667) | 0.708 | 0.479 | 0.098  (0.230) | 0.425 | 0.671 | -0.0139  (0.287) | -0.0484 | 0.961 |

Legend: rG(SE)=genetic correlation estimate and the standard error, Z score=rG/SE

**Supplementary Table 2**. Detailed results of the Mendelian randomization results between adiposity phenotypes and pancreatic cancer

| **Exposure** | **Outcome** | **MR Method** | **NSNPs** | **OR(95%CI)** | **P value** | **Q statistic**  **(P value)** |
| --- | --- | --- | --- | --- | --- | --- |
| BMI | PanC | MR Egger | 566 | 0.999 (0.997-1.001) | 0.389 | 500.41 (0.974) |
| BMI | PanC | Weighted median | 566 | 1.000 (0.999-1.002) | 0.561 | NA |
| BMI | PanC | Inverse variance weighted | 566 | 1.001 (1.000-1.001) | 0.090 | 502.99 (0.971) |
| BMI | PanC | Simple mode | 566 | 1.001 (0.997-1.004) | 0.714 | NA |
| BMI | PanC | Weighted mode | 566 | 1.000 (0.998-1.002) | 0.802 | NA |
| WHRadjBMI | PanC | MR Egger | 278 | 1.001 (0.999-1.0032) | 0.268 | 258.035 (0.774) |
| WHRadjBMI | PanC | Weighted median | 278 | 1.0012 (0.9998-1.0027) | 0.095 | NA |
| WHRadjBMI | PanC | Inverse variance weighted | 278 | 1.00095 (1.00011-1.0018) | 0.027 | 258.078 (0.787) |
| WHRadjBMI | PanC | Simple mode | 278 | 0.9998 (0.997-1.0032) | 0.927 | NA |
| WHRadjBMI | PanC | Weighted mode | 278 | 1.0009 (0.9987-1.0031) | 0.417 | NA |
| PanC | BMI | MR Egger | 16 | 0.444 (0.000-11023.55) | 0.877 | 99.368 (6.27x10-15) |
| PanC | BMI | Weighted median | 16 | 58.105 (3.997-844.69) | 0.003 | NA |
| PanC | BMI | Inverse variance weighted | 16 | 58.526 (0.301-11367.20) | 0.130 | 108.025 (3.86x10-16) |
| PanC | BMI | Simple mode | 16 | 70.019 (3.66-1341.18) | 0.013 | NA |
| PanC | BMI | Weighted mode | 16 | 91.921 (7.73-1092.50) | 0.003 | NA |
| PanC | WHRadjBMI | MR Egger | 16 | 21.142 (0.00-64574354.85) | 0.695 | 32.171 (3.79x10E-03) |
| PanC | WHRadjBMI | Weighted median | 16 | 0.070 (0.000-74.001) | 0.454 | NA |
| PanC | WHRadjBMI | Inverse variance weighted | 16 | 0.143 (0.000-222.403) | 0.604 | 33.487 (4.018x10-03) |
| PanC | WHRadjBMI | Simple mode | 16 | 1.057 (0.000-11211.818) | 0.991 | NA |
| PanC | WHRadjBMI | Weighted mode | 16 | 0.137 (0.000-99.21) | 0.563 | NA |

Legend: PanC=pancreatic cancer, NSNPs=number of SNPs/genetic instruments used to estimate causality, OR(95%CI)=Odds ratio and the lower and upper 95% confidence intervals (CI).
